# The effect of strontium ranelate on titanium particle-induced periprosthetic osteolysis regulated by WNT/β-catenin signaling *in vivo* and *in vitro*

**DOI:** 10.1042/BSR20203003

**Published:** 2021-01-29

**Authors:** Bolun Wang, Haohui Guo, Tianxiang Geng, Kening Sun, Liang Zhang, Zhidong Lu, Qunhua Jin

**Affiliations:** 1 Department of Orthopedic Surgery, Ningxia Medical University, 1160 Shengli Street, Xingqing Area, Yinchuan, Ningxia, P.R. China 750004; 2 Department of Orthopedics, General Hospital of Ningxia Medical University, 804 Shengli Street, Xingqing Area, Yinchuan, Ningxia, P.R. China 750004

**Keywords:** Strontium ranelate, Peri-prosthetic osteolysis, Wear particles, Osteogenesis, WNT/β-catenin signaling

## Abstract

Aseptic loosening following periprosthetic osteolysis is the primary complication that limits the lifetime of total joint arthroplasty (TJA). The wear particles trigger a chronic inflammation response in the periprosthetic tissue and turn over the bone balance to bone resorption. The present study aimed to investigate the possible effect and mechanism of strontium ranelate (SR), a clinically safe drug for osteoporosis, on particle-induced periprosthetic osteolysis. Thirty-six female C57BL/6j mice underwent tibial Ti-nail implantation to establish an animal model of aseptic loosening. After 12 weeks, micro-CT results showed that strontium ranelate could inhibit periprosthetic bone resorption. *In vitro*, Ti particles were used to stimulate RAW264.7 cell line to collect conditioned medium, and co-culture MC3T3-E1 cell line with conditioned medium to establish a cell model of aseptic loosening. The results of alkaline phosphatase (ALP) detection, immunofluorescence, and flow cytometry demonstrated that strontium ranelate could regulate the expression of OPG/RANKL, promote differentiation and mineralization, and inhibit apoptosis in osteoblasts. Moreover, we revealed that SR’s exerted its therapeutic effect by down-regulating sclerostin, thereby activating the Wnt/β-catenin signal pathway. Therefore, this research suggests that strontium ranelate could be a potential drug for the prevention and treatment of particle-induced aseptic loosening post-TJA.

## Impact statement

Over recent years, the number of cases of primary TJA has increased, revealing that progressively younger patients require surgery. The prevention of aseptic loosening and prolongation of the lifetime of prostheses are the major problems post-TJA. Compared with new surgical techniques or new prosthetic materials, oral medications are cost-effective, quick-acting, especially for patients who underwent primary TJA. Herein, we proved the effectiveness of strontium ranelate, a clinically safe drug, in preventing and treating aseptic loosening. The results indicated that strontium ranelate could promote osteoblasts by activating the Wnt/β-catenin signal pathway to inhibit particle-induced periprosthetic osteolysis. Thus, strontium ranelate provides a new, acceptant, and economical method for treating aseptic loosening post-TJA.

## Introduction

Total joint arthroplasty (TJA) can effectively alleviate the pain caused by various joint diseases, and reconstruct the damaged joint function especially for the end-stage arthropathy. TJA is known as one of the most significant breakthroughs in orthopedics in the twentieth century. The long-term postoperative follow-up visits revealed a 20-year survival rate higher than 80% [1,2]. Many complications restrict the lifetime of the prosthesis implanted in TJA. At the same time, aseptic loosening induced by wear particles has been identified as the primary cause for postoperative failure, accounting for approximately 75% of revision cases [3]. Over recent years, the number of patients with postoperative aseptic loosening has increased [4]. There is no effective treatment method other than complicated revision surgery. The previous studies suggested that the biological reaction between periprosthetic tissues and wear particles induces chronic inflammation, which in turn brakes the bone metabolic balance and leads to periprosthetic osteolysis [5,6].

Researchers have conducted many studies to prevent and treat aseptic loosening, which focused on the improvement of materials, enhancement of techniques, or application of medication [7,8]. The drug research mainly focused on how to inhibit osteoclasts and reduce the expression of inflammatory factors [7,9]. Drugs such as erythrocin, bisphosphonates, herbal, or denosumab have been proven to block osteoclast formation or antagonize the inflammatory factors [10,11]. However, it is impossible to apply new materials or techniques specific to a loosened prosthesis. On the other hand, pharmacological studies mainly center on inhibiting osteoclasts or decreasing inflammatory factors. Nevertheless, these medicines have particular side effects or have not undergone clinical trials yet [12]. Therefore, a clinically proven safe drug that could simultaneously inhibit bone resorption and promote bone formation would be useful for treating aseptic loosening.

Strontium ranelate (SR) is a drug that has been clinically used in the treatment of osteoporosis, which has shown to be especially effective for postmenopausal women [13]. Daily oral administration of 2g SR may effectively decrease the risk of vertebral fracture in postmenopausal osteoporosis patients [14]. Clinical trials have shown that SR could promote bone growth, increase bone density, and reduce the risk of spinal, peripheral, or hip fractures [15]. Moreover, SR has been proven to increase bone formation and decrease bone resorption at the same time, and transform bone balance to bone formation [16–18]. Besides, Liu et al. demonstrated that SR could inhibit osteoclastogenesis and regulate the inflammatory response induced by wear particles in the mouse calvaria resorption model [19]. However, the role of SR in aseptic loosening of TJA and its possible effect on osteoblasts remain unclear.

The aim of the present study was to investigate the role of SR in wear particle-induced osteolysis *in vivo* and *in vitro*. We hypothesized that SR could inhibit particle-induced periprosthetic osteolysis and promote the differentiation and maturation of osteoblasts by regulating the Wnt signaling pathway. In order to verify our hypothesis, the mice were given low or high-concentration SR via intra-gastric administration for 12 weeks post tibial Ti-nail implantation, followed by micro-CT and histopathological assays. On the other hand, RAW264.7 and MC3T3-E1 cell lines were used to establish a wear particle-induced osteolytic cell model, and ALP activity assay, immunofluorescence, and flow cytometry were employed to discover the function and mechanism of SR. We hypothesized that the application of SR would have a better outcome and compliance after TJA. In addition, aseptic loosening was expected to become a new indication for this safe and economical drug.

## Methods

### Preparation of titanium particles

Commercial titanium (Ti) particles were purchased from Alfa Aesar, Thermo Fisher Scientific (Heysham, Lancashire, U.K.). According to the certificate of analysis, the mean particle diameter was 2.92 μm. More than 93% of the particles were <20 μm, 50% were <1.6 μm, and 10% were <1.0 μm, which is the clinically relevant size range [20]. Ti particles were incubated at 180°C for 6 h prior to being washed by 75% ethanol four times for 1 h to eliminate endotoxins and then immersed in dehydrated ethanol overnight in the dark [21]. The endotoxin level was evaluated using the Limulus Amebocyte Lysate Assay (Lonza, Walkersville, MD, U.S.A.). Microscopic observation of Ti particles was carried out by scanning electron microscope (S-3400N, HITACHI, Tokyo, Japan). Particles were resuspended with sterile phosphate-buffered saline (PBS); they were vortexed each time and sonicated before use [22].

### Animal experiment and drug treatment

Thirty-six 10-week-old female C57BL/6j mice (Experimental Animal Center of Ningxia Medical University, Yinchuan, China), each weighing 20 ± 2 g, were used in the present study. All the mice were housed with pressure-controlled ventilation at 25°C and 40–70% humidity in a 12/12h-light/dark cycle, and were given lab chow and water ad libitum. The experimental protocol was conducted under the National Institutes of Health guidelines for the care and use of laboratory animals [23] and approved by the Ethics Committee of General Hospital of Ningxia Medical University.

The murine model of prosthesis failure was performed as previously described [24]. Briefly, mice were subjected to knee prosthesis implantation in the right tibia under general anesthesia induced by intraperitoneal injection of Nembutal (0.6% pentobarbital sodium; Neobioscience, Shenzhen, China). Under the aseptic condition, the proximal tibia condyle was exposed via the medial parapatellar approach, and a cavity for the implant was reamed with a 1.0 mm dental drill in the center of the tibia plateau. A Ti nail (diameter, 1.2 mm; length, 5 mm) was press-fitted into the cavity, in a way that the surface of the nail-head was flush with the cartilaginous surface of the tibial plateau. The wound was washed with normal saline containing 100 U/ml penicillin and 100 μg/ml streptomycin, and each layer was separately closed with absorbable sutures [24]. For the induction of osteolysis, mice were injected with 10 μl Ti suspension (approximately 4 × 10^4^ particles) into the tibial cavity before the insertion of Ti nail. Moreover, 20 μl Ti particle suspension was intra-articularly injected every 2 weeks following the surgery.

Mice were randomly divided into three groups for treatment with SR (Protelos; Servier, Stoke Poges, U.K.) as Control group (surgery only), Low-SR group (surgery and 625 mg kg^−1^day^−1^ SR), and High-SR group (surgery and 1800 mg kg^−1^day^−1^ SR). All groups were given SR or equal water via intragastric gavage for 12 weeks and then were sacrificed in a 0.2–0.5% carbon monoxide box for histological, immunohistochemical, and micro-computed tomography analysis.

### Micro-computed tomography analysis

The tibiae of four mice per group were harvested and fixed in 4% paraformaldehyde solution after removing soft tissues at 4°C for 1 week. The fixed tibiae specimens were scanned using micro-computed tomography (μCT, SkyScan 1176; Bruker, Kontich, Belgium) with parameters set at 9 μm per layer, 45 kW voltage, 550 mA current, 900 ms exposure [25]. Reconstruction and analysis of the acquired images were conducted by the manufacturer’s software (NRecon ver.1.1, Bruker) after scan to assess bone volume fraction (BV/TV), trabecular thickness (Tb.Th), trabecular bone number (Tb.N), and the bone surface area around the Ti nail (BS/BV) [25]. The cross-sectional image was taken at half of the Ti nail as 2 mm to the lower edge of the nail cap.

### Histological and immunochemical analysis

The tibiae of four mice per group were fixed in 4% paraformaldehyde solution at 4°C for 24 h. The specimens were then immersed in 10% EDTA solution for 3 weeks of decalcification. All samples were dehydrated by the gradient concentration of ethanol and xylene and then dipped in paraffin at 60°C for 10 min. Paraffin-coated specimens were cut at 5 μm per section perpendicular to the long axis of the tibia using a handy microtome RM2235 (Leica Microsystems; Leitz, Wetzlar, Germany). The sections were analyzed for histological morphology using hematoxylin and eosin (H&E) staining. Briefly, the specimens were stained by 0.5% eosin solution for 5 min, following by hematoxylin solution for 3 min at room temperature.

Additionally, the sections were analyzed by immunohistochemical staining for the expression of osteoprotegerin (OPG) and nuclear factor-κB receptor activating factor ligand (RANKL) to evaluate the osteolytic disruption around the Ti nail. Briefly, the sections were immersed in a 60°C EDTA solution for 2 min of antigen retrieval. After rinsing with PBS, the specimens were incubated in 3% hydrogen peroxide at room temperature for 10 min, and then the primary antibody was added dropwise on the slide for overnight incubation. The concentrations and species of primary antibodies were rabbit polyclonal anti-mouse OPG 1:200 (ab183910; Abcam, Cambridge, U.K.) and rabbit polyclonal anti-mouse RANKL 1:500 (ab9957; Abcam). To exclude non-specific reaction, a negative control was performed using PBS instead of primary antibody. Afterward, labeled goat anti-rabbit IgG polymer (Sino Biological, Beijing, China) was used as the secondary antibody for 40 min at room temperature. Finally, sections were imaged using an upright optical microscopy imaging system (DM2000, Leica), and positive expression results were calculated using Image-Pro Plus 6.0 (Media Cybernetics; Rockville, MD, U.S.A.).

### Cell culture

The mouse preosteoblast cell line MC3T3-E1 (ATCC, CRL-2593) was cultured with α-MEM medium (Gibco, Thermo Fisher Scientific) containing 10% fetal bovine serum (FBS), 100 U/ml penicillin and 100 μg/ml streptomycin (Gibco) at a 37°C, 5% CO_2_ humid incubator and medium was changed every 2 days. The mouse monocyte/macrophage cell line RAW264.7 (ATCC, TIB-71) was cultured with DMEM medium (Gibco) containing 10% FBS, 100 U/ml penicillin and 100 μg/ml streptomycin at a 37°C, 5% CO_2_ humid incubator and the medium was changed every day. When the cells were grown to 80–90% confluency, 0.25% trypsin was added to the culture flask for digestion after washing by PBS, and the adherent cells were passaged at a ratio of 1:3.

### Collection of conditioned media

RAW264.7 cells were seeded with DMEM medium at a density of 1.0 × 10^5^/60 mm dish. The conditioned media were collected according to a previously described procedure with minor modifications [26]. In brief, after adherence for 24 h, the cells were washed with sterile PBS and stabilized with a serum-free DMEM medium for 1 h. At that time, the titanium particles, which have been disinfected and endotoxin-degraded, were added to the culture dish, and the ratios of cells to Ti particles are 1:10, 1:50, 1:100, or 1:200. Also, the control group was added to the same medium volume with sterile PBS. After 6, 12, or 24 h of stimulation, the culture medium of the control group (named Cont CM) and Ti particles stimulation group (named Ti CM) were separately collected. The cell debris in the medium was removed by centrifugation at 2500 ***g***, and the conditioned media were stored at −20°C for further research. Meanwhile, tartrate-resistant acid phosphatase (TRAP) staining was performed using a commercial TRAP kit (Sigma-Aldrich, Merck KGaA, Darmstadt, Germany) to observe the phagocytosis of RAW264.7 cells after stimulation with Ti particles.

### Cell Counting Kit-8 Assay

The cytotoxicity of SR was determined using Cell Counting Kit-8 (CCK-8, Transgen Biotech, Beijing, China). According to the manufacturer’s protocol, MC3T3-E1 cells were harvested and adjusted to the density at 1.0 × 10^5^/ml, inoculating into a 96-well cell culture plate at 200 μl per well. After cell adherence, different concentrations (0.1–5.0 mM) of SR (MedChemExpress, NJ, U.S.A.) were added for 48 h of incubation, and triplicate wells were set in each group. Finally, 20 μl CCK-8 solution was added to each well, and incubation was continued for another 4 h at 37°C, then each well was measured by a multi-plate reader BioTek Synergy 2 (Winooski, VT, U.S.A.). The effect of SR on the cell viability of MC3T3-E1 was evaluated according to OD_450_ value. The experiment was repeated three times, and the averaged value was calculated for each concentration.

### Alkaline phosphatase activity assay

Alkaline phosphatase is a marker enzyme of mature osteoblasts that catalyzes the hydrolysis of p-nitrophenyl phosphate (pNPP) to p-nitrophenol, which is yellow and has a maximum absorption peak at 405 nm. According to the previously reported method [27], the ALP assay kit (Millipore, Merck KGaA) was used to analyze the ALP activity of MC3T3-E1 cells. In a 24-well cell culture plate (9.0 × 10^4^/well), the mixed medium containing 50% α-MEM media and 50% conditioned media was added to MC3T3-E1 cells after adherence, in which the SR was set to three different concentrations (0.1, 0.5, and 1.0 mM). After incubation for 48 h, the cells were washed twice with cold PBS, and 0.2 ml RIPA buffer was used to lyse cells for 30 min. Then, the assay buffer and pNPP substrate (1.5 mg/ml) was added, and the cells were incubated at 37°C for another 30 min. The OD_405_ value was measured using a microplate reader. The total protein of each well was measured using a BCA Protein Assay Kit (KeyGen BioTECH, Jiangsu, China).

### Osteoblast differentiation and Alizarin Red S staining

For differentiation of MC3T3-E1, 50 mg/ml ascorbic acid, 10 nM dexamethasone, and 10 mM β-glycerophosphate (Sigma-Aldrich) were added in the α-MEM medium [28]. After 14 days of osteogenic medium culture, the mineralized matrix deposition was analyzed by Alizarin Red staining. The cells were fixed with 4% paraformaldehyde for an hour, and then incubated with 40 mM alizarin red staining working solution (Cyagen, Suzhou, China) for 30 min at room temperature. The stained cells were observed under an optical microscope.

### Annexin V-FITC/PI apoptosis detection by FCM

To investigate the inhibitory effect of SR on osteoblasts apoptosis, we stained MC3T3-E1 with Annexin V-FITC/PI and detected fluorescence by flow cytometer. The FITC Annexin V Apoptosis Detection Kit was purchased from BD Biosciences (San Diego, CA, U.S.A.), and the cells treated with Ti CM and SR were collected by trypsin digestion according to the manufacturer’s protocol. In brief, cells were sequentially stained with FITC Annexin V or PI working solutions at room temperature in the dark for 15 min. Then 1× binding buffer was added, and at least 1.0 × 10^4^ cells per group were collected by flow cytometer (BD Accuri C6 Plus, New Jersey, U.S.A.) to quantify the apoptosis rate of osteoblasts. Different subgroups were classified: Q1 annexin V negative, PI-positive (i.e., necrotic cells); Q2, annexin V/PI double-positive (i.e., dead cells); Q3, annexin V positive, PI negative (i.e., apoptotic cells); Q4, annexin V/PI double-negative (i.e., live cells).

### Immunofluorescence and confocal laser scanning microscopy assay

To detect and locate the expression changes of nuclear factor кB receptor activating factor ligand (RANKL) and osteoprotegerin (OPG) under the intervention of Ti CM and SR in osteoblasts, we performed cellular immunofluorescence combined with confocal laser microscopy assay. The cells were grouped and treated as described above. Afterward, the cells were fixed with 4% paraformaldehyde for 10 min and then permeabilized with 0.1% Triton X-100 for 20 min at room temperature. Non-specific binding sites were blocked by normal donkey serum. The cells were incubated with rabbit anti-mouse RANKL (ab9957, Abcam, 1 μg/ml) and goat anti-mouse OPG (AF459, R&D systems, 1 μg/ml) antibody overnight at 4°C, after which they were washed three times with PBS and incubated with donkey anti-rabbit IgG H&L (Alexa Fluor 488, ab150073, Abcam, 1:500) and donkey anti-goat IgG H&L (Alexa Fluor 555, ab150130, Abcam, 1:500) fluorescent secondary antibodies for 30 min at 37°C. Finally, the cells were counterstained with DAPI in the dark and photographed under 40-fold using a confocal laser microscope system (FV-1000, Olympus, Japan).

### RNA isolation and quantitative real-time PCR analysis

Total RNA was extracted using E.Z.N.A. total RNA Kit I (Omega Bio-Tek, Norcross, GA, U.S.A.) according to the manufacturer’s protocol, and the purity of RNA was measured by 260/280 absorbance ratio. In brief, RNA was extracted by spin column and reverse-transcribed to cDNA using RevertAid First Strand cDNA Synthesis Kit (Thermo Scientific). Real-time PCR was performed by SYBR Premix Ex Taq II (Takara) and Bio-Rad CFX real-time PCR detection system (Hercules, CA, U.S.A.). The reaction conditions were set as denaturation at 95°C for 15 s, amplification at 60°C for 30 s, and 40 cycles repeat. All PCR samples were analyzed with triplicate wells, and glycerol-3-phosphate dehydrogenase (GAPDH) was used as the internal control. The relative expression level of mRNA was calculated using the -2^ΔΔCt^ method [29]. The mouse primer sequences synthesized by Sangon Biotech (Shanghai, China) are shown in Supplementary Table S1.

### Luciferase reporter assay

MC3T3-E1 was seeded at a density of 2 × 10^4^/well in 96-well plate, and cells were transfected with plasmid using Lipofectamine 3000 (Invitrogen, Thermo Fisher Scientific) to construct a luciferase reporter gene system. TopFlash luciferase reporter (Addgene Plasmid #12456, Cambridge, MA, U.S.A.) (100 ng) and Renilla luciferase thymidine kinase vector (Invitrogen) (50 ng) were used to detect the activation of WNT/β-catenin signaling in MC3T3-E1 cells [30]. A dual-luciferase assay kit (Promega, Madison, WI, U.S.A.) was used as previously described [26], and the luminescence of luciferase was measured by the microplate reader Synergy 2 (BioTek). Triplicate wells were set in all samples and normalized to the Renilla luciferase activity.

### Activation and inhibition of WNT/β-catenin signal pathway

To further verify whether SR regulates osteoblasts via the WNT/β-catenin signal pathway, the WNT signaling was activated or inhibited. For activation, 5 ng/ml recombinant mouse Wnt-3a protein (R&D systems) was added to the above-described culture system of MC3T3-E1, and the luciferase activity of cells was measured after 48 h of co-treatment with SR. For inhibition, 5 μM ICG-001 (MedChemExpress), which specifically binds to cyclic AMP response element-binding protein, was used to antagonize WNT/β-catenin/TCF transcription [31]. The inhibitory treatment and cell luciferase activity assay were identical to the activation treatment by Wnt-3a.

### Protein isolation and Western blotting

MC3T3-E1 cells were seeded at 3 × 10^5^/well in six-well cell culture plate. After 48 h of treatment with Ti CM and SR, cells were washed twice with cold PBS and then lysed with RIPA buffer containing protease inhibitor and phosphatase inhibitor. The lysates were centrifuged, and the supernatant was collected to determine the protein concentration using a BCA Protein Assay Kit (KeyGEN BioTECH). Total cell protein was separated by 12% SDS-PAGE and then transferred to a PVDF membrane. After 2 h of blocking with the Tris-tween buffer containing dry milk, samples were incubated with the primary antibody overnight at 4°C. Primary antibodies used in the present study were β-actin (1:1000), RANKL (1:1000), OPG (1:500), sclerostin (SOST, 1:1000) (Abcam), β-catenin (1:1000) (Cell Signaling Technology, MA, U.S.A.). Finally, the membrane was incubated with horseradish peroxidase-conjugated secondary antibody (ZSGB-bio, Beijing, China) for 2 h at room temperature. The protein on the membrane was detected as a fluorescent band by the enhanced chemiluminescence reagent (KeyGEN BioTECH).

### Enzyme-linked immunosorbent assay (ELISA)

Titanium particles (1:10,1:50,1:100) were added to RAW264.7 cells for 12 or 24 h of stimulation, respectively. The Ti CM or Cont CM was collected after stimulation, and the content of inflammatory cytokines in Ti CM or Cont CM was quantitatively determined using the mouse TNF-α, IL-1β, and IL-6 ELISA Kit (Neobioscience, Shenzhen, China) according to the manufacturer’s protocol.

### Statistical analysis

All data were recorded as mean ± standard deviation (SD) and assessed by the Kolmogorov–Smirnov test to ensure normality before statistical analysis. One-way analysis of variance (ANOVA) and Student–Newman–Keuls test were used for multiple comparisons. *P*<0.05 were considered as statistically significant. All statistical analyses and diagrams were performed by GraphPad Prism 7.0 software (San Diego, CA, U.S.A.).

## Results

### Characterizations of titanium particles and nail

The scanning electron microscopy revealed that the titanium particles were of a polygonal shape and different sizes. Photographs were taken at 500 or 5000 times magnification (Supplementary Figure S1A). The results showed that the average size of Ti particles was 2.92 ± 1.30 μm, with 93% of the particles being <20 μm, 50% <1.6 μm, and 10% <1.0 μm (data not shown). A titanium nail was implanted in the murine knee joint to simulate arthroplasty (Supplementary Figure S1B). The general condition of mice after the operation was good and showed no significant rejection of implants (Supplementary Figure S1C).

### Effect of strontium ranelate on periprosthetic osteolysis *in vivo*

**Figure 1:**
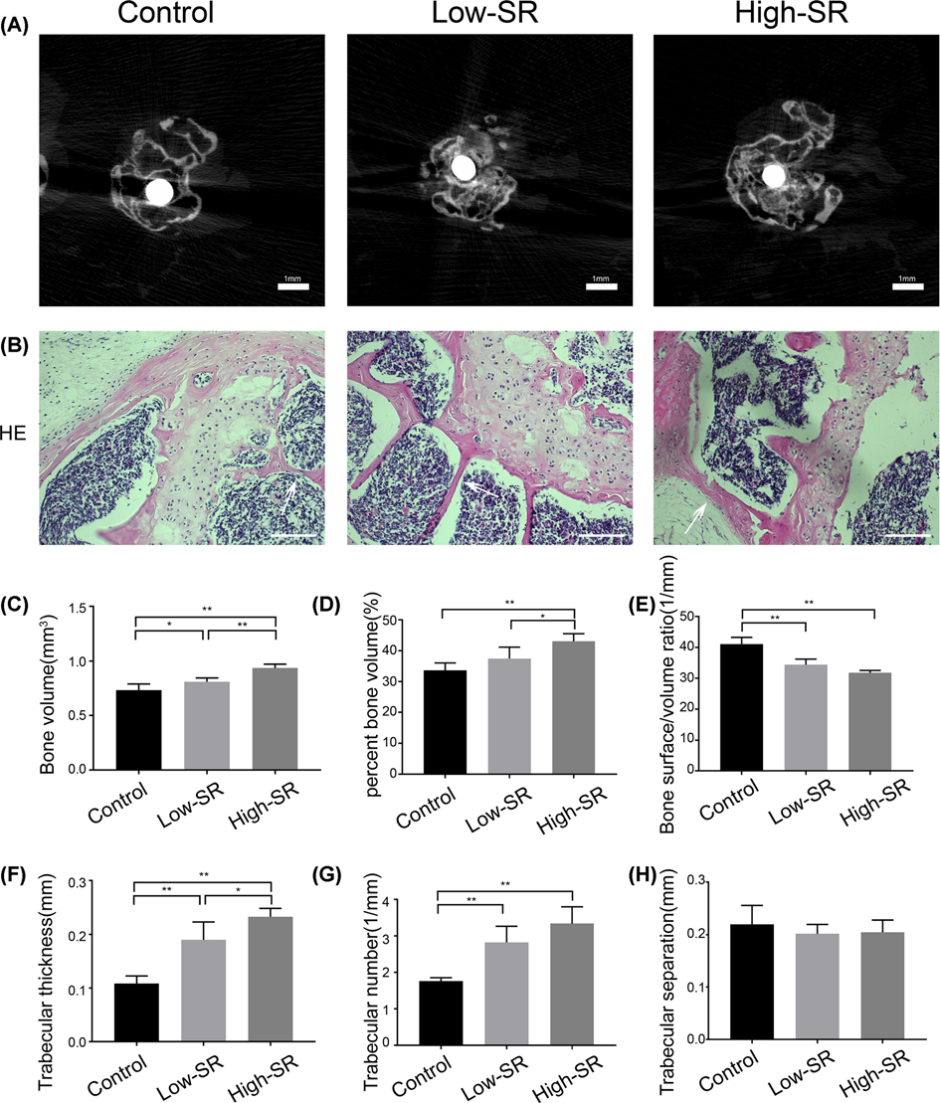
Effect of strontium ranelate on periprosthetic osteolysis in mice (**A**) Representative micro-CT fault images of the murine tibia (scale bar = 1 mm). (**B**) Hematoxylin and eosin staining (scale bar = 100 μm). (**C**) Bone volume. (**D**) Bone volume fraction. (**E**) Bone surface/volume ratio. (**F**) Trabecular thickness. (**G**) Trabecular number. (**H**) Trabecular separation. Low-SR means 625 mg/kg/day and High-SR means 1800 mg/kg/day (*P<0.05 and **P<0.01).

Micro-CT scan, which was conducted after 12 weeks, revealed the periprosthetic osteolysis and a destroyed bone structure in the control group, with a significant defect of cancellous bone. In the SR treatment group, osteolysis was relatively minor, with less bone defect. The effect of inhibiting osteolysis in the high-dose group was superior to the low-dose group (Figure 1A). Analysis of bone tissue related parameters showed that treatment with SR could significantly enhance the bone volume (Figure 1C) and bone volume fraction (Figure 1D) around the prosthesis, meanwhile decrease the bone surface per unit volume (Figure 1E). The bone volume of 0.731 ± 0.057 mm^3^ increased to 0.936 ± 0.034 mm^3^, and bone volume fraction increased from 33.71% ± 2.35% to 43.06% ± 2.48%, while bone surface density decreased from 41.12 ± 2.18 to 31.83 ± 0.77 l/mm. SR also showed its effect on the trabecular growth, trabecular thickness (Figure 1F), and quantity (Figure 1G), which were significantly increased. The trabecular thickness of the high-dose group (0.232 ± 0.016 mm) was higher than the low-dose group (0.190 ± 0.033 mm), while the difference in trabecular quantity between the two groups was not significant. Moreover, the difference of trabecular separation between the SR groups and the control group was not statistically significant (Figure 1H). The specific analysis data are shown in Table 1. A similar result was also found in HE staining of bone tissue (Figure 1B). These results suggested that SR could inhibit periprosthetic osteolysis and promote osteogenesis.

**Table 1:** Micro-CT analysis data of bone tissue (*n* = 6 per group)

	Control	Low-SR	High-SR
BV (mm^3^)	0.731 ± 0.057	0.808 ± 0.035*	0.936 ± 0.034†
BV/TV (%)	33.71 ± 2.35	37.49 ± 3.68	43.06 ± 2.48†
BS/BV (1/mm)	41.12 ± 2.18	34.49 ± 1.75†	31.83 ± 0.77†
Tb.Th (mm)	0.108 ± 0.014	0.190 ± 0.033†	0.232 ± 0.016†
Tb.N (1/mm)	1.761 ± 0.097	2.825 ± 0.444†	3.341 ± 0.460†
Tb.Sp (mm)	0.219 ± 0.036	0.202 ± 0.018	0.204 ± 0.023

*P<0.05, †*P*<0.01, compared with Control group; BS/BV, bone surface area density; BV, bone volume; BV/TV, bone volume fraction; Tb.N, trabecular number; Tb.Th, trabecular thickness; TB.Sp, trabecular separation.

### Suppressed ALP activity by Ti CM *in vitro*

**Figure 2:**
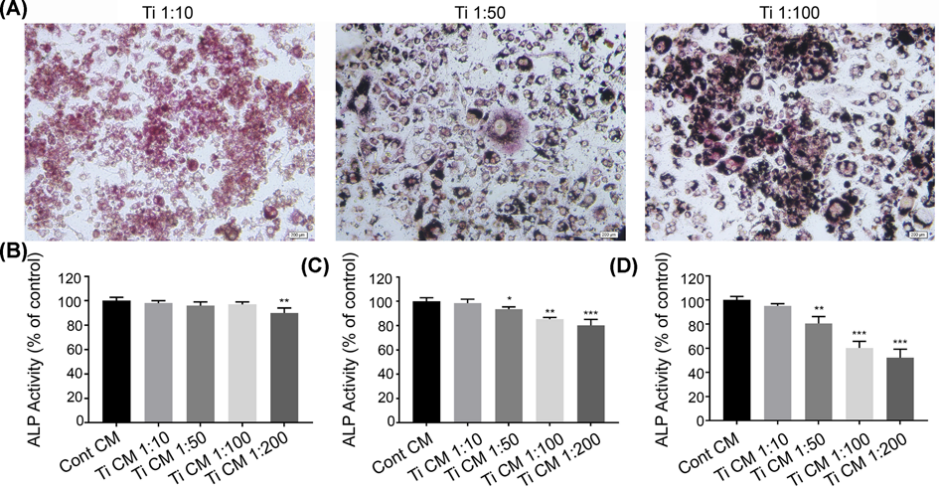
Conditioned media suppressed ALP activity in osteoblasts (**A**) TRAP staining for RAW264.7 after Ti particles stimulation 24 h (scale bar = 200 μm). (**B**) ALP activity after stimulation 6 h. (**C**) ALP activity after stimulation 12 h. (**D**) ALP activity after stimulation 24 h. Ti 1:10 means the number of Ti particles is ten times to cells (*P<0.05, **P<0.01, and ***P<0.001).

To further explore the effect of SR on periprosthetic osteolysis induced by wear particles, we established an osteolysis model *in vitro*. TRAP staining for RAW264.7 cells showed that phagocytosis occurred under the stimulation with Ti particles at three variable proportions, in which groups of 1:50 and 1:100 were found with cells devouring Ti particles, while the cellular morphology of 1:10 group did not significantly change (Figure 2A). Meanwhile, the content of inflammatory factors in Ti CM or Cont CM quantified by ELISA revealed that RAW264.7 cells could secrete a large amount of TNF-α, IL-1β, and IL-6 under the stimulation. The specific content is shown in Table 2.

**Table 2:** Concentrations of TNF-α, IL-1β, and IL-6 in each conditioned media

	Cont CM	Ti CM		
		1:10	1:50	1:100
TNF-α (ng/ml)	1.67 ± 0.15	1.62 ± 0.09	2.52 ± 0.21†	4.24 ± 0.32†
IL-1β (ng/ml)	1.04 ± 0.24	1.17 ± 0.19*	1.25 ± 0.11*	1.65 ± 0.15†
IL-6 (ng/ml)	9.43 ± 1.32	9.28 ± 1.55	11.34 ± 1.67*	12.75 ± 2.20*

Data are shown as the mean ± SD, measured by ELISA. Compared with Cont CM group, *P<0.05

and †*P*<0.01. IL-1β, interleukin-1β; IL-6, interleukin-6; TNF-α, tumor necrosis factor-α.

Alkaline phosphatase is an iconic enzyme of mature osteoblasts. The co-culturing of MC3T3-E1 cells was conducted by mixing α-MEM medium with Ti CM or Cont CM, and ALP activity was detected after 24 h. In the groups stimulated by Ti particles for 6 h, the ALP activity of 1:200 significantly declined (Figure 2B), while in the 12 h (Figure 2C) and 24 h (Figure 2D) groups, ALP activities of 1:50, 1:100, and 1:200 all significantly decreased. The osteoblastic ALP activity of 1:100 group decreased to 60.23% ± 5.47% after 24 h of stimulation as compared with the control group. Thus, we selected Ti CM obtained by 24 h stimulation at Ti 1:100 proportion for subsequent study.

### The therapeutic effect of strontium ranelate on inflammatory osteoblasts

**Figure 3:**
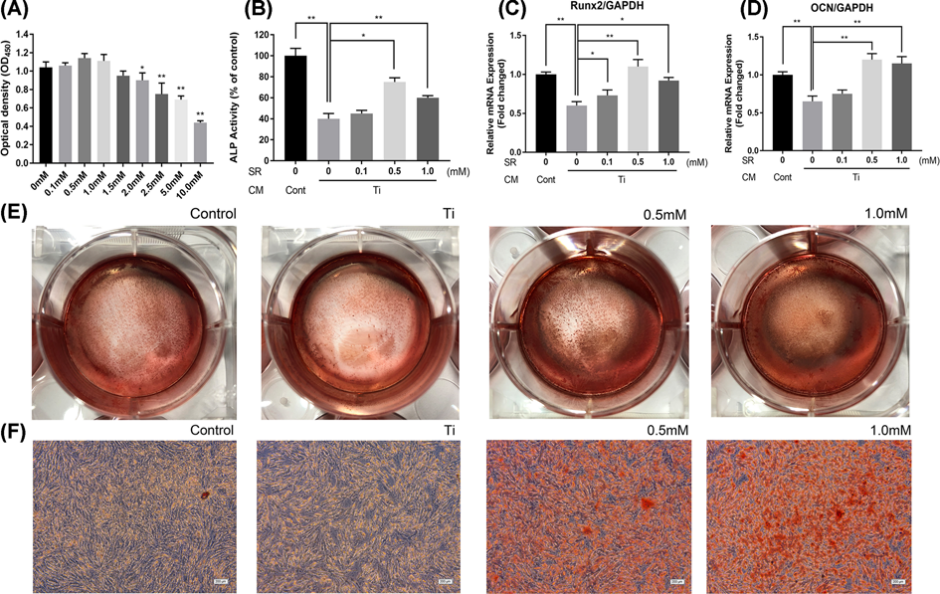
Therapeutic effect of strontium ranelate on inflammatory osteoblasts (**A**) MC3T3-E1 was treated with various concentrations of SR. After 48 h, the CCK-8 test was performed. (**B**) ALP activity of MC3T3-E1 after co-culturing with conditioned media and SR. (**C**) Relative mRNA expression of Runx2. (**D**) Relative mRNA expression of OCN. (**E**) Alizarin red staining for MC3T3-E1 after differentiation culture. (**F**) Alizarin red staining images under a microscope (scale bar = 200 μm; *P<0.05 and **P<0.01)

SR was added in the osteolysis model established as above reported to explore the effect on osteoblasts under the inflammatory environment. CCK-8 test was performed to determine the cytotoxicity induced by SR, and the result showed that SR could induce a significant decline in cell viability when its concentration was higher than 2.0 mM (Figure 3A). Therefore, we designed three concentrations of SR for study, i.e., 0.1, 0.5, and 1.0 mM. After the addition of SR in the co-culture system for 48 h, the result demonstrated that 0.5 and 1.0 mM SR could increase the reduced ALP activity induced by Ti CM from 40.23% ± 5.50% to 75.11%± 4.71% and 60.02% ± 2.39%, respectively (Figure 3B). We also detected the marker genes related to osteoblasts maturation. RT-PCR results revealed that after treatment with SR for 48 h, mRNA expressions of Runx2 in the SR groups were elevated (Figure 3C), among which 0.5 mM group was the most significant (1.10 ± 0.09-fold). The increased mRNA expression of OCN was presented in 0.5 mM (1.20 ± 0.08-fold) and 1.0 mM (1.15 ± 0.09-fold) groups except for 0.1 mM group (Figure 3D). These results showed the short-term effect of SR on osteoblasts. On the other hand, alizarin red staining was applied to evaluate the long-term treatment of SR on MC3T3-E1 with 14 days’ differentiation culture. Results demonstrated that cell density in the Ti CM group was lower than in the control group, while cell aggregation was found in the SR treatment groups with a red dye attached (Figure 3E). Similarly, mineralized nodules with red staining were found under a microscope in 0.5 and 1.0 mM SR groups (Figure 3F). The above results proved that SR has a therapeutic effect on maturation and differentiation in osteoblasts.

### Effect of strontium ranelate on the expression of OPG and RANKL

**Figure 4:**
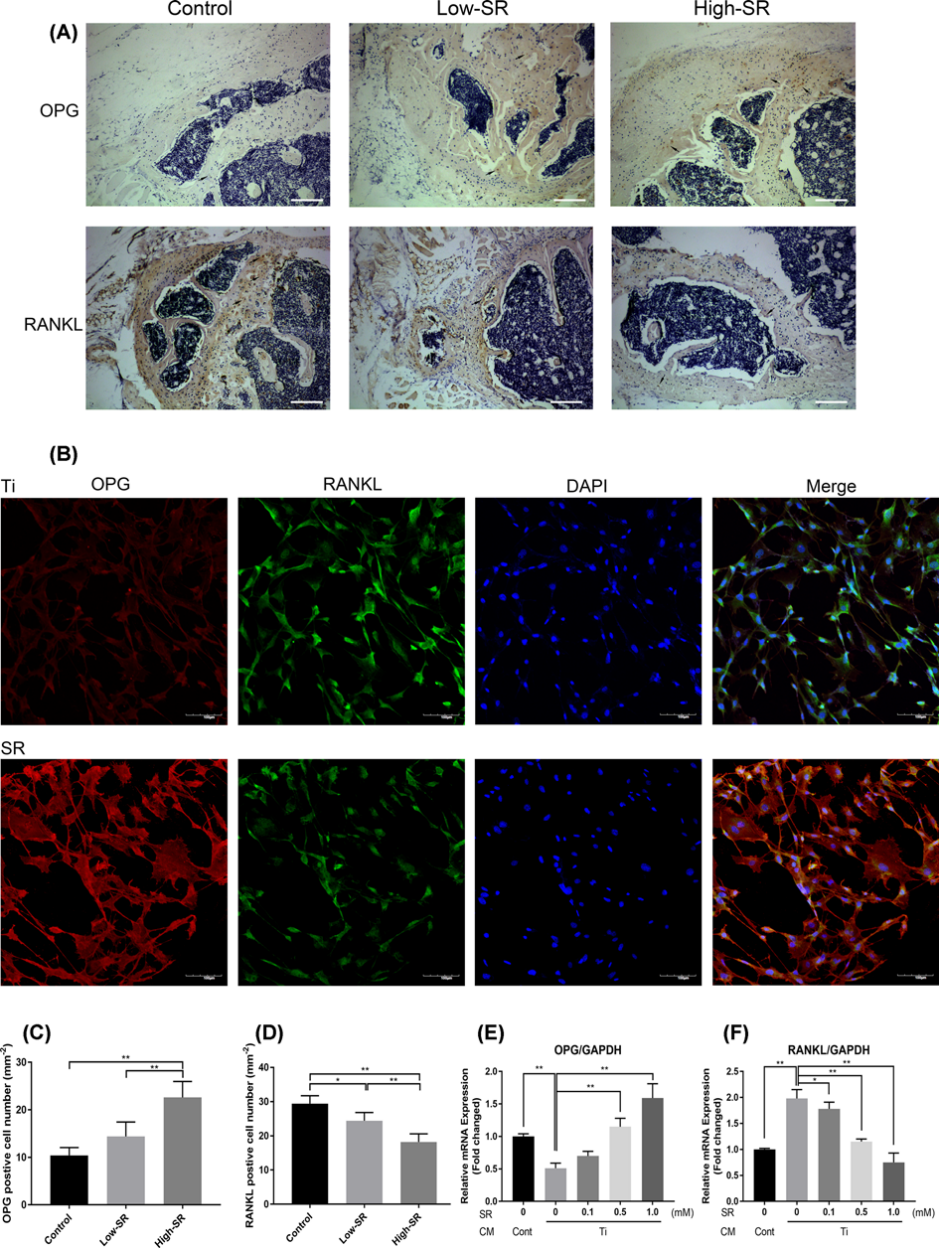
Strontium ranelate regulated the expression of OPG/RANKL in vivo and in vitro (**A**) OPG and RANKL immunohistochemical staining images (scale bar = 100 μm). (**B**) Cell immunofluorescence images of Ti CM and 1.0 mM SR groups (scale bar = 100 μm). (**C**) OPG positive cell numbers. (**D**) RANKL positive cell numbers. (**E**) Relative mRNA expression of OPG. (**F**) Relative mRNA expression of RANKL (*P<0.05 and **P<0.01).

RANKL/OPG/RANK axis has a vital role in osseous cells and bone metabolism [32]. After the mice received SR for 12 weeks, the immunohistochemical staining was applied to detect RANKL and OPG. The results showed that the control group had few OPG positive cells, but a large number of cells with positive RANKL expression. In the SR groups, cells with positive OPG expression increased as compared with the control group, while the number of positive RANKL cells decreased (Figure 4A). The number of positive cells in ROI was counted by software, and the results showed that the number of OPG positive cells in the high-dose SR group was higher than the control group, while there was no difference in the low-dose group (Figure 4C). In contrast, the numbers of RANKL positive cells in both low-dose and high-dose SR groups were statistically lower than in the control group (Figure 4D).

Immunofluorescence double staining was used to detect the expression and localization in *in vitro* osteolysis model. The results were reconfirmed using an *in vivo* experiment. Red fluorescence represents OPG protein, green fluorescence represents RANKL protein, and blue fluorescence represents cell nucleus. After merging OPG, RANKL, and DAPI fluorescence images, a similar result was presented (Figure 4B). Meanwhile, further confirmation of the immunofluorescence result was conducted by RT-PCR, and the mRNA expression showed that SR could up-regulate OPG expression (Figure 4E) and down-regulate RANKL expression (Figure 4F).

### Effect of strontium ranelate on Ti CM-induced osteoblasts apoptosis

**Figure 5:**
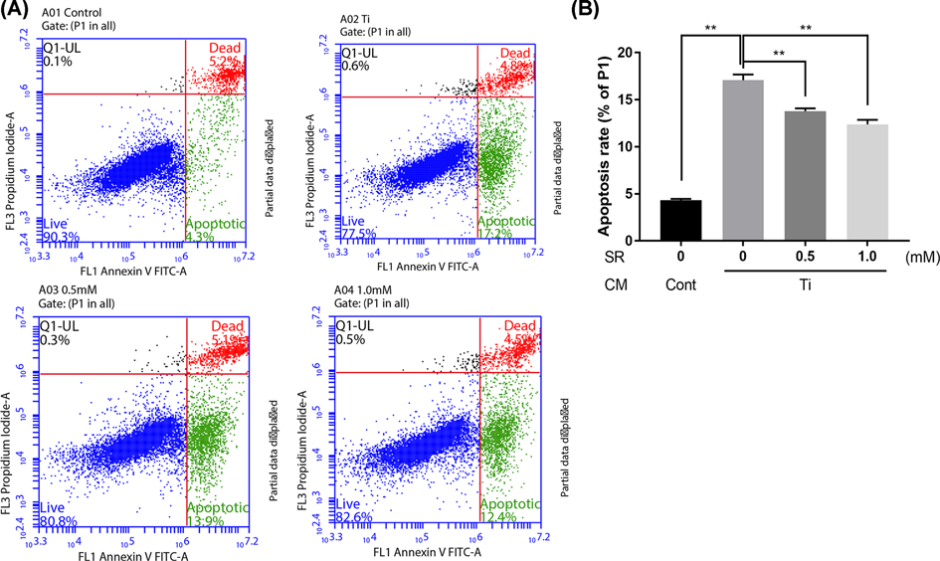
Strontium ranelate suppressed Ti CM-induced apoptosis of osteoblasts (**A**) The scatter plots of flow cytometry. (**B**) Apoptosis rate of osteoblasts in each group (**P<0.01).

To clarify the effect of SR on osteoblast apoptosis, FITC fluorescence-labeled Annexin V and PI were used to double stain MC3T3-E1. The cells at different apoptosis stages were detected and classified by a flow cytometer (Figure 5A). The results demonstrated that the cell apoptosis rate of MC3T3-E1 with Ti CM co-culturing for 24 h increased from 4.33% ± 0.13% to 17.07% ± 0.60%. While the apoptosis rates of 0.5 and 1.0 mM SR groups were significantly decreased (13.77% ± 0.30% and 12.38% ± 0.48%, respectively; Figure 5B). The above outcomes showed that co-culturing with Ti CM might lead to osteoblast apoptosis, while application of 0.5 or 1.0 mM SR might significantly suppress the apoptosis and recover cell viability.

### Effect of strontium ranelate on WNT/β-catenin signal pathway

**Figure 6:**
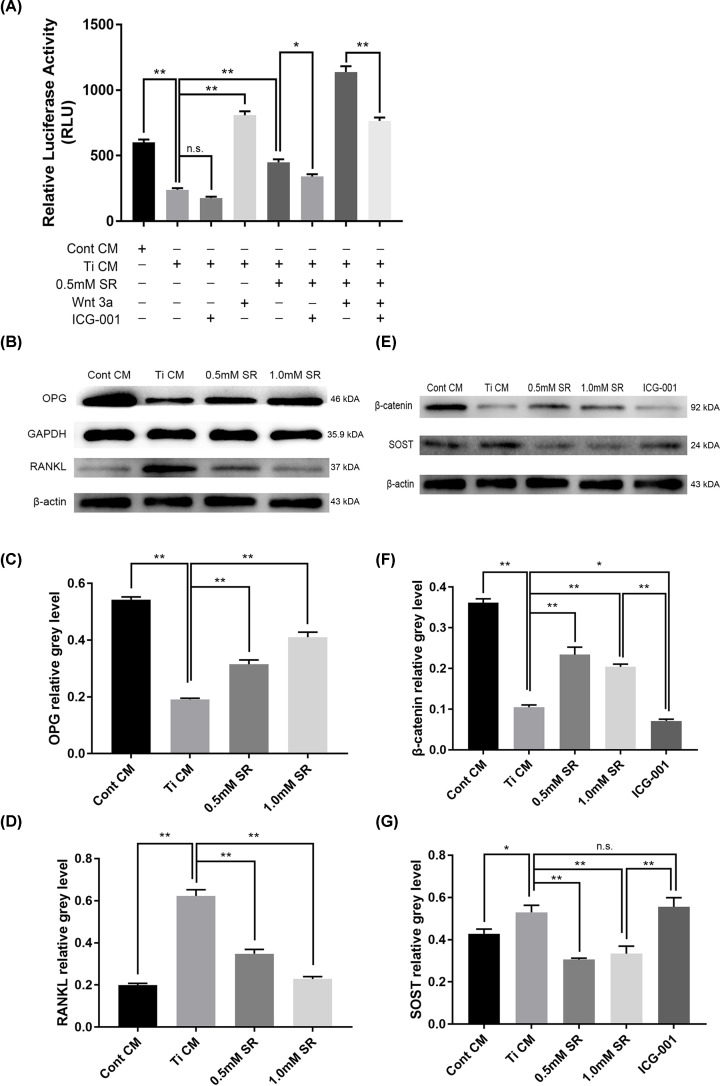
Strontium ranelate activated Wnt/β-catenin signal pathway by downregulating sclerostin in osteoblasts (**A**) Relative luciferase activity in each group (+ means presence, - means absence). (**B** and **E**) After the intervention of Ti CM for 48 h, total osteoblasts lysates were collected and subjected to detect proteins by Western blotting. (**C**) OPG relative grey level. (**D**) RANKL relative gray level. (**F**) β-Catenin relative gray level. (**G**) SOST relative gray level. (n.s. = no significance, *P<0.05 and **P<0.01)

To further investigate the possible mechanism of SR on osteoblasts, the TOPFlash dual-luciferase reporter gene system was established to reflect the transcription of WNT/β-catenin signaling. After the co-culturing of Ti CM for 48 h, TopFlash luciferase activity in osteoblasts declined from 601.4 ± 22.06 RLU to 238.9 ± 13.02 RLU (approximately 0.39-fold), while in 0.5 mM SR group, the luciferase activity was 449.0 ± 22.65 RLU, showing the increase of approximately 1.80-fold compared with Ti CM group. Moreover, after adding ICG-001 to both groups, luciferase activity in the Ti CM group declined to 177.0 ± 9.91 RLU (*P*>0.05), and the SR group declined to 340.1 ± 17.89 RLU (*P*<0.05), indicating that blockage of WNT/β-catenin signaling affected the effect of SR on osteoblasts. Besides, the luciferase activity in the group that added both SR and recombinant Wnt 3a reached the peak, while it was inhibited after adding ICG-001 (Figure 6A).

Finally, Western blotting was used to detect the expression of RANKL, OPG (Figure 6B), β-catenin, and SOST (Figure 6E) in each group, and GAPDH or β-actin were used as internal controls. The results showed that OPG expression declined by about 0.35-fold under the intervention of Ti CM, while after the application of SR, OPG expression was restored (Figure 6C). On the contrast, Ti CM up-regulated RANKL expression approximately 3.13-fold, which could be inhibited by SR (Figure 6D). Furthermore, we detected the effect of SR on β-catenin and SOST. The results proved that compared with the control group, Ti CM down-regulated the β-catenin expression by approximately 0.29-fold, while both 0.5 and 1.0 mM SR could significantly promote β-catenin expression, which was inhibited by adding ICG-001 (Figure 6F). Also, the expression of SOST was in contrast with β-catenin. Ti CM facilitated a small rise of SOST, which was inhibited by the application of SR. However, in the ICG-001 group, the inhibitory effect of SR was weakened, and SOST expression showed no difference compared with the Ti CM group (Figure 6G). The above results revealed that SR could activate WNT/β-catenin signaling by down-regulating SOST expression in order to promote osteoblasts.

## Discussion

As a listed medication that was applied years ago in the treatment of osteoporosis, SR can effectively lower the risk of bone fracture in postmenopausal female patients [15,33]. In the present study, we discovered that SR might inhibit *in vivo* and *in vitro* periprosthetic osteolysis induced by wear particles. SR is a strontium (Sr^2+^) salt of ranelic acid, where both Sr and Ca elements belong to the IIA group in the periodic table of elements. Previous studies have demonstrated that Sr^2+^ might facilitate osteogenesis and inhibit interleukin, which has been applied in artificial biological materials [34,35]. In the present study, we established a mouse tibial Ti-nail implantation model and injected Ti particles into the articular cavity to mimic aseptic loosening *in vivo*. Our results showed that SR might effectively increase BV, BV/TV, and BS/BV in periprosthetic tissue after 12 weeks. Also, the quantity and thickness of trabecula were significantly increased, while trabecular separation did not change. Our *in vivo* result was consistent with that reported by Liu et al. in a mouse calvaria resorption model [19]. By contrast, our tibial implantation model might better mimic the process of aseptic loosening in patients. However, our model was not flawless in terms of complicated surgery and late occurrence of osteolysis. The above results revealed that SR might inhibit particles induced chronic periprosthetic osteolysis in mice and slow down the occurrence of aseptic loosening.

The macrophage has an essential role in the pathological process of aseptic loosening. There is a large number of macrophages in the interface between the prosthesis and bone tissue [36], which can devour particles of different materials with a diameter below 10 mm and release multiple inflammatory factors into the periprosthetic microenvironment [36,37]. To mimic this process, we used the RAW264.7 monocyte line and MC3T3-E1 osteoblast line to establish an *in vitro* cell model of aseptic loosening. Our results showed that Ti CM obtained by stimulation of 24 h at 1:100 proportion might effectively reduce ALP activity in osteoblasts. Meanwhile, the ELISA detection demonstrated that Ti CM contained a large number of inflammatory factors, such as TNF-α, IL-1β, and IL-6. The modeling results in the present study were similar to that of Lee et al. [26] but different with reference to the optimal particle proportion and stimulation time, which may be due to different particle preparation methods or cell activity. Moreover, we conducted TRAP staining on RAW264.7 to observe the process of cells phagocytosing Ti particles as well as cells aggregating.

According to the RCT research, daily oral administration of 2 g SR for osteoporosis females could reduce vertebral fractures by 37% and nonvertebral fractures by 14% over 3 years [15]. We provided intragastric administration for mice at 625 and 1800 mg kg^−1^d^−1^ SR for 12 weeks, which was generally similar to previous animal studies of SR, where researchers set two concentration groups [19,38]. For *in vitro* studies, investigators applied different doses specific to different cell lines. For example, Vidal et al. used SR to interfere with mesenchymal stem cells, where 1–2 mM did not generate a significant effect [39]. However, in the study by Li et al., 0.1 mM SR could inhibit the proliferation of stem cells [40]. Consequently, we utilized the CCK-8 test to determine the cytotoxicity of SR, showing that at concentrations higher than 2.0 mM, SR could significantly inhibit osteoblasts activity. The concentration varied in cell study, which may attribute to different reactions of cell lines to SR.

Osteoblasts are the crucial cells for the maintenance of bone metabolism, which are mainly responsible for bone formation during the pathological process of periprosthetic osteolysis [41]. After co-culturing with metal, polymethyl methacrylate (PMMA), or ultrahigh molecular weight polyethylene (UHMWPE) particles, osteoblasts revealed reduced expression of procollagen α1 gene and type I collagen [42,43]. In the present study, we discovered that after co-culturing with Ti CM, ALP activity of osteoblasts was significantly declined, similarly to the transcription of Runx2 and OCN, thus indicating that osteogenesis was inhibited. In contrast, the inhibited osteogenesis by Ti CM was restored after the application of SR. ALP, a hydrolase widely distributed in tissues and organs, is secreted from gallbladder via the liver. Moreover, its type III isoenzyme comes from osteocytes as a marker of mature osteoblast [44]. In the present study, we observed the early effect of SR on osteoblasts; however, periprosthetic osteolysis is a continuous process of aseptic inflammation. Patients post joint replacement face the risk of periprosthetic osteolysis for a lifetime. Besides, MC3T3-E1 is an osteogenic precursor cell line with particular differentiation potential [45]. For these reasons, we conducted an osteogenic differentiation culture for MC3T3-E1 in 14 days. The staining result presented that SR could facilitate the differentiation of osteoblasts and aggregate cells to form mineralized nodules on long-term. For the first time, we built a cell model composed of differentiation medium, SR and Ti CM to mimic the inflammatory environment of osteoblasts during periprosthetic osteolysis. We also proved that SR might promote proliferation, differentiation, and mineralization of osteoblasts *in vitro*.

RANKL is known as a membrane protein secreted by osteoblasts, which could activate osteoclasts and facilitate bone resorption [46,47]. Besides, OPG is a decoy receptor to RANKL, which can compete with RANK to bind RANKL to inhibit osteoclastogenesis and bone resorption [48,49]. Osteoblasts secrete RANKL/OPG in a state of dynamic equilibrium, affected by microenvironment or cell stage. RANK/RANKL/OPG axis is a critical pathway to maintain the symbiotic relationship between bone resorption and bone formation [32,50]. In the present study, IHC staining results in mice showed that OPG positive cells in the SR group were increased, while RANKL positive cells were decreased. The expression and distribution of OPG and RANKL in osteoblasts were observed *in vitro* by using a multi-channel laser confocal microscope. A similar result was also found in mRNA expression of OPG and RANK in osteoblasts. The above *in vivo* and *in vitro* experiments proved that SR inhibits particle-induced osteolysis by regulating the expression of RANKL/OPG in osteoblasts.

Another possible explanation for the protective effect of SR on osteoblasts is the inhibition of apoptosis induced by particles. Apoptosis is a programmed cell death that occurs in multicellular organisms, which is mainly internally regulated by cell, while the external microenvironment or signals may also activate apoptosis [51]. In this research, we used Annexin V-FITC/PI dual-staining osteoblasts and detected the apoptosis rate by flow cytometry. Statistical results revealed that osteoblast apoptosis rate increased about three times under the intervention of Ti CM, which significantly decreased after the application of SR. Different from other TJA complications caused by bacteria infection or open injury, aseptic loosening is a chronic inflammatory response caused by particles, in which apoptosis is more common [52–54]. On the other hand, as osteoblasts mature, RANKL secretion gradually decreases, while OPG expression increases [55]. However, a shortcoming of our study is the absent detection of apoptosis-related factors; thus, future studies should address factors such as caspase and Bcl-2.

Wnt signaling is a set of signal transduction pathways. Currently, three pathways are known to be activated by binding Wnt ligand with the frizzled receptor, which transmits biological signals to disheveled protein inside cells [56]. Wnt signal was first recognized for its role in tumorigenesis and its ability to control embryonic development and tissue regeneration of bone marrow, skin, and intestine [57]. Over recent years, researchers have discovered that the Wnt/β-catenin signaling pathway is crucial in bone metabolism. For example, bone marrow mesenchymal stem cells can differentiate into osteoblasts and secrete type I collagen by activating the Wnt pathway [58]. Also, during the process of osteoblasts development, the Wnt pathway can prolong cell life [59]. Therefore, we constructed a dual-luciferase reporter gene system to detect transcription of Wnt/β-catenin signal pathway. Under the intervention of Ti CM, the transcription of Wnt signaling was inhibited, which was resumed after adding SR. In addition, we used the Wnt pathway-specific inhibitor ICG-001 to block β-catenin mediated transcription [60]. As a result, the effect of SR was suppressed, while alone application of ICG-001 brought no difference in luciferase activity. Moreover, the WB results showed that SR might improve the synthesis of β-catenin, an essential protein downstream in the Wnt signaling [61]. In contrast, the synthesis was reduced with the addition of ICG-001. Particularly, we found for the first time that SR may down-regulate the expression of sclerostin in osteoblasts. Sclerostin is a secretory glycoprotein whose domain and sequence are similar to the DAN family [62]. Sclerostin is a product of the SOST gene, which was initially considered as a nonclassic BMP antagonist. Over recent years, it has been found that it can inhibit Wnt signaling by binding to LRP5/6 receptor [63]. In brief, we proposed that SR may inhibit particle-induced osteolysis by down-regulating SOST, thereby activating the Wnt signal pathway.

The present study has some limitations that need to be pointed out. First, we used Ti particles as wear debris instead of ultrahigh molecular weight polyethylene, which are commonly found in aseptic loosening [64]. The reason is that Ti particles are more accessible than PE particles, and metal particles are stable for sterilization by autoclave. Meanwhile, previous studies have reported that metal particles are equivalent to polyethene particles in terms of inducing periprosthetic osteolysis [20]. Another limitation is that the *in vivo* model in the present study was aseptic loosening in the knee, without considering possibilities of other joints, especially hip joint, in which aseptic loosening also tends to frequently occur [4]. Nevertheless, compared with the conventional mouse calvaria resorption model, the model we used takes mechanical stress and wear into consideration; however, it also has some disadvantages such as complicated surgery.

Over recent years, the number of TJA cases increased, revealing that progressively younger patients were undergoing surgery [65]. It has also been estimated that the demand for primary total hip/knee arthroplasties will reach 4 million by 2030 in the U.S.A. [66]. In the meantime, the rise of technical improvements such as porous-coated prosthesis or computer design customized prosthesis has reduced the incidence of aseptic loosening [67,68]. However, the high cost of new technologies and the learning process for surgeons have limited widespread promotion. Oral medications have the advantages of low cost, quick effect, and secure promotion, especially for patients who received primary TJA, which can prolong the lifetime of prostheses and delay revision. In the present study, we investigated SR, a marketed drug for osteoporosis, to verify its inhibitory effect and possible mechanisms on particle-induced periprosthetic osteolysis. In one RCT, SR was associated with an increased risk of venous thromboembolism [69], while another clinical trial reported that patients with cardiovascular disease and uncontrolled hypertension occasionally experienced adverse outcomes after using SR [70]. Consequently, in our subsequent studies on the pharmacological mechanism and clinical trials, we will exclude patients with cardiovascular risk factors. Moreover, patients with low bone mineral density, especially postmenopausal female patients with osteoporosis, will be selected for further research that will also evaluate the risk-benefit of long-term use of SR.

In conclusion, our study demonstrated that strontium ranelate, a clinically proven safe drug for osteoporosis, inhibits particle-induced periprosthetic osteolysis by promoting osteogenesis *in vivo* and *in vitro*. Moreover, we found that SR could regulate the expression of OPG and RANKL in osteoblasts while inhibiting apoptosis induced by Ti particles and activate the Wnt/β-catenin signal pathway by down-regulating sclerostin. These findings indicated that strontium ranelate could be used as a potential drug for the prevention and treatment of aseptic loosening post-TJA.

## Supplementary Material

Supplementary Figure S1 and Table S1

## Data Availability

All supporting data are included within the main article and its supplementary files. For any other data for this paper, anyone can get access by email us.

## Abbreviations

GAPDH: glycerol-3-phosphate dehydrogenase
OPG: osteoprotegerin
PMMA: polymethyl methacrylate
RANKL: nuclear factor кB receptor activating factor ligand
SR: strontium ranelate
TJA: total joint arthroplasty
TRAP: tartrate-resistant acid phosphatase
