# Supplementary material for: The effect of strontium ranelate on titanium particle-induced periprosthetic osteolysis regulated by WNT/β-catenin signaling in vivo and in vitro
Source: Biosci Rep. 2021 Jan 29;41(1):BSR20203003. doi: 10.1042/BSR20203003 (PMC7846966; doi:10.1042/BSR20203003)

Table S1. Primers used for RT-PCR

Target	Forward primer (5'–>3')	Reverse primer (3'–>5')	GenBank No.
Runx2	F:CCTCTGACTTCTGCCTCTGG	R:ATGAAATGCTTGGGAACTGC	NM_009820.5
OCN	F:CCATCTTTCTGCTCACTCTGC	R:ACCTTATTGCCCTCCTGCTT	NM_007541.3
OPG	F:GAGAAGCCACGCAAAAGTGT	R:GGCAAACTGTCCACCAAAAC	NM_008764.3
RANKL	F:AGGAGGGAGCACGAAAAACT	R:AAGGGTTGGACACCTGAATG	NM_011613.3
SOST	F:CCTCATCTGCCTACTTGTGC	R:ATGGTCTGGTTGTTCTCAGG	NM_024449.6
GAPDH	F:ACCCAGAAGACTGTGGATGG	R:CACATTGGGGGTAGGAACAC	NM_008084.3

Note! *Runx2: runt-related transcription factor 2; OCN: osteocalcin; OPG: osteoprotegerin; RANKL: receptor activator of unclear factor kappa-B ligand; SOST: sclerostin; GAPDH: glyceraldehydes 3-phosphate dehydrogenase.*

Fig.S1 Titanium particles and titanium nail used in this study. (A) Scanning electron microscope images x500 (Scar bar = 100μm) or x5000 (Scar bar = 10μm). **(B)** Ti nail. **(C)** X-ray image of murine knee after surgery (Scar bar = 1.6mm), **(D)** skeletal formula of strontium ranelate.

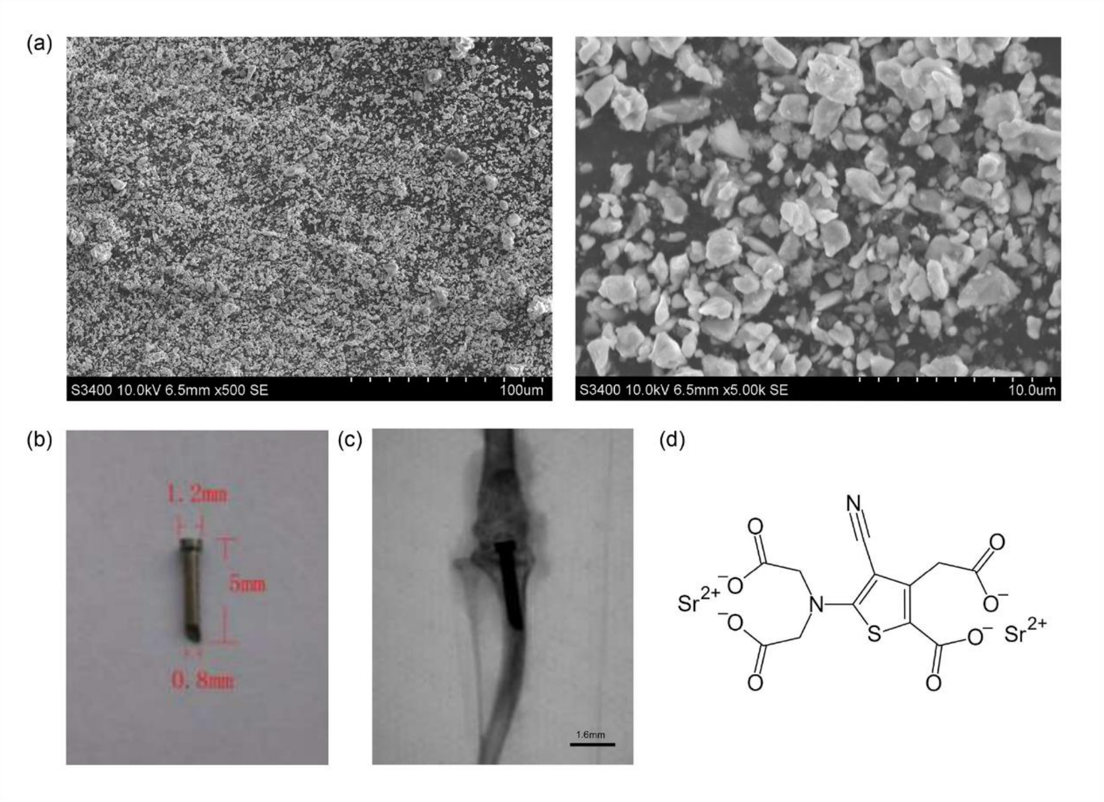

Supplement: Supplementary Figure S1 and Table S1 [file BSR-2020-3003_supp.pdf]
